# Associations between parental type 2 diabetes risk and offspring birthweight and placental weight: a survival analysis using the Walker cohort

**DOI:** 10.1007/s00125-022-05776-5

**Published:** 2022-08-11

**Authors:** Carlos Sánchez-Soriano, Ewan R. Pearson, Rebecca M. Reynolds

**Affiliations:** 1 Centre for Cardiovascular Science, Deanery of Molecular, Genetic and Population Health Sciences, Queen’s Medical Research Institute, University of Edinburgh, Edinburgh, UK; 2 Division of Population Health and Genomics, Ninewells Hospital and School of Medicine, University of Dundee, Dundee, UK

**Keywords:** Birthweight, Fine–Gray regression, Intergenerational associations, Placental weight, Survival analysis, Type 2 diabetes, Walker cohort

## Abstract

**Aims/hypothesis:**

Low birthweight (BW) is associated with the development of type 2 diabetes. Genome-wide analyses have identified a strong genetic component to this association, with many BW-associated loci also involved in glucose metabolism. We hypothesised that offspring BW and placental weight (PW) are correlated with parental type 2 diabetes risk, reflecting the inheritance of diabetes risk alleles that also influence fetal growth.

**Methods:**

The Walker cohort, a collection of birth records from Dundee, Scotland, from the 1950s and the 1960s was used to test this hypothesis by linking BW and PW measurements to parental health outcomes. Using data from SCI-Diabetes and the national death registry, we obtained health records for over 20,000 Walker parents. We performed Fine–Gray survival analyses of parental type 2 diabetes risk with competing risk of death, and Cox regression analyses of risk of death, independently in the maternal and paternal datasets, modelled by offspring BW and PW.

**Results:**

We found significant associations between increased paternal type 2 diabetes risk and reduced offspring BW (subdistribution hazard ratio [SHR] 0.92 [95% CI 0.87, 0.98]) and PW (SHR 0.87 [95% CI 0.81, 0.94]). The association of maternal type 2 diabetes risk with offspring BW or PW was not significant. Lower offspring BW was also associated with increased risk of death in both mothers (HR 0.91 [95% CI 0.89, 0.94]) and fathers (HR 0.95 [95% CI 0.92, 0.98]), and higher offspring PW was associated with increased maternal mortality risk (HR 1.08 [95% CI 1.04, 1.13]) when adjusted for BW.

**Conclusions/interpretation:**

We identified associations between offspring BW and reduced paternal type 2 diabetes risk, most likely resulting from the independent effects of common type 2 diabetes susceptibility alleles on fetal growth, as described by the fetal insulin hypothesis. Moreover, we identified novel associations between offspring PW and reduced paternal type 2 diabetes risk, a relationship that might also be caused by the inheritance of diabetes predisposition variants. We found differing associations between offspring BW and PW and parental risk of death. These results provide novel epidemiological support for the use of offspring BW and PW as predictors for future risk of type 2 diabetes and death in mothers and fathers.

**Graphical abstract:**

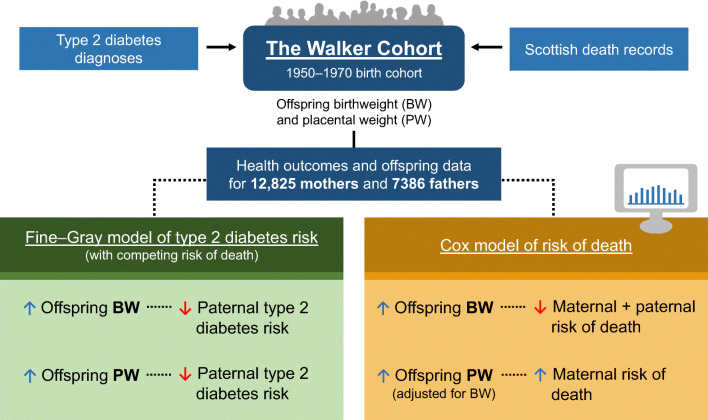

**Supplementary Information:**

The online version of this article (10.1007/s00125-022-05776-5) contains peer-reviewed but unedited supplementary material..

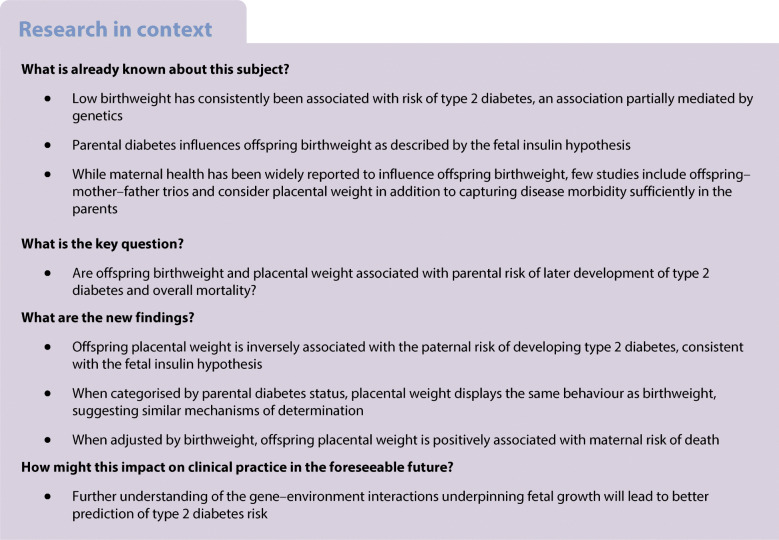



## Introduction

Observational studies have identified consistent associations between low birthweight (BW) and type 2 diabetes mellitus in multiple independent populations [1, 2]. It has been hypothesised that this association is genetically mediated, with both low BW and type 2 diabetes susceptibility being phenotypes of the same genotype [3, 4]. According to the fetal insulin hypothesis, polymorphisms influencing glucose metabolism (and thus later diabetes) are also associated with impaired fetal growth, through the actions of insulin. In fact, multiple loci associated with BW at genome-wide significance levels are also inversely genetically correlated with traits such as type 2 diabetes, fasting glucose and fasting insulin during later life [5, 6]. Maternal type 2 diabetes risk alleles also influence this relationship, affecting intrauterine glucose homeostasis and increasing offspring BW [6–8].

Despite the progress made unravelling the relationship between early development and diabetes, the great majority of epidemiological studies investigating the link between parental diabetes and offspring birth outcomes involve only mother–offspring pairs. Few studies include fathers [9–13], which allows the study of the effect of paternal variants expressed by the fetus, independently of the glycaemic status of mothers. In addition, placental weight (PW) is seldom considered, despite being the tissue on which fetal growth depends. Further, retrospective studies usually involve a compromise between the quality of and detail in birth records and length of follow-up. Many studies using older cohorts rely on self-reported birth measurements, and individuals unresponsive or lost to follow-up may introduce selection bias.

The Walker cohort [14] is a collection of exhaustive birth records from the 1950s and 1960s, based in Dundee, Scotland. The cohort was set up in this industrialised urban region after the post-World War II rationing. During nearly 20 years, over 48,000 hospital births (75% of all births in Dundee for that period) were meticulously documented by clinical staff, who recorded a multitude of measurements such as BW and PW directly onto birth cards. These cards also included information on the babies’ mothers and fathers, allowing the identification of full family trios. Through data linkage, more than 70 years’ worth of longitudinal healthcare information can be obtained for the Walker parents, a population with significant type 2 diabetes morbidity and overall mortality. The Walker cohort represents a valuable dataset from which to draw insights into the association between fetal growth and parental disease. Because of the previously reported genetic link between type 2 diabetes and BW, we hypothesised that offspring BW and PW would be phenotypically associated with the parental risk of developing type 2 diabetes through the inheritance of type 2 diabetes risk alleles. To investigate these associations, survival models of parental risk of type 2 diabetes diagnosis (adjusted for the competing risk of death) and risk of death, modelled by offspring BW and PW, were built independently for the mothers and fathers included in the Walker cohort.

## Methods

### Study population and data sources

All individuals were part of the Walker cohort [14]. Offspring BW, PW, gestational age (GA) and sex were obtained from the Walker records (1951–1968), completed at the time of birth by obstetricians. When unavailable (73% of individuals), GA was inferred from the time between the last menstrual period and the date of birth (DOB). If last menstrual period information was not available, GA was inferred from the time between 280 days before the recorded estimated delivery date and the actual DOB. Parental information was obtained by linkage to additional datasets using Community Health Index numbers, NHS Scotland’s unique identifiers. Walker parents and offspring diagnosed with type 2 diabetes were identified (and their dates of diagnosis obtained) through SCI-Diabetes [15]. Any identifiable individuals not present in the SCI-Diabetes extract were defined as not having been diagnosed with any type of diabetes. Parental DOB and health board-specific Scottish Index of Multiple Deprivation (HBSIMD, 2019 v2 release), which categorises areas according to their deprivation quintile (from 1, most deprived, to 5, least deprived), were obtained from the national Community Health Index dataset. Parental date of death was obtained from the National Records of Scotland.

### Data exclusions

Individuals with a GA <37 weeks or ≥43 weeks, BW <2.5 kg or >5.5 kg or PW <200 g or >1000 g were excluded from the analysis so that only healthy term pregnancies were included. An additional set of analyses performed with wider inclusion ranges (GA <37 or ≥43 weeks, BW <900 g or >6.5 kg, PW <200 g or >1500 g) to include all term births is shown in the electronic supplementary material (ESM). Only singleton pregnancies and firstborn child births were included. To compare the effects of offspring BW and PW as explanatory variables for the survival analysis, and take into account any variation due to offspring sex, both variables were sex stratified and standardised through *Z* transformation. The analyses included all identifiable individuals living in the area (Tayside) since 1 January 1986, when type 2 diabetes diagnosis data were routinely collected. The study endpoint was defined as the date of the last type 2 diabetes diagnosis event (22 November 2020). The datasets for the analyses included 12,825 mothers and 7386 fathers, 84.6% and 84.5% of the total identifiable Walker parents, respectively.

### Statistical analyses

All analyses were conducted using R statistical software [16] version 3.6.2.

#### Summary statistics

The cumulative percentages of Walker mothers and fathers who developed type 2 diabetes or died were calculated, removing individuals who had left the Tayside Health Board. Summary tables of diabetes status and death by 10-year periods were built separately for the living identifiable mothers and fathers. Welch’s two-sample unpaired *t* tests were used to investigate differences between the offspring of parents with and without type 2 diabetes. The comparison of variables of interest by *Z*-transformed offspring BW and PW quartiles was performed using information up to 2010, to maximise the sample of living parents and as type 2 diabetes prevalence was similar to the national 2012 diabetes estimates for over 65 year olds in Tayside [17]. The difference in variables between quartiles was tested through ANOVA, using the mean value for each quartile. In the case of type 2 diabetes diagnosis and death, the calculated percentages were used. The barplots comparing BW and PW by parental type 2 diabetes status were built using only offspring whose mothers and fathers could be identified (*n*=6469).

#### Survival analysis study design

The association between offspring BW and PW and parental type 2 diabetes diagnosis risk was investigated through survival analysis. Because of the age of the population in the study, the risk of death was stipulated as competing with the risk of type 2 diabetes diagnosis, using a Fine–Gray regression model [18]. The parental risk of death was investigated through Cox regression [19]. Mothers and fathers were studied separately. Three survival endpoint events were defined for the model. Individuals who left the area during the study time frame and individuals alive at the end of the study without having been diagnosed with type 2 diabetes were coded as censored (32.7% of mothers, 20.1% of fathers). Individuals who developed type 2 diabetes were coded as event 1 (15.3% of mothers, 17.1% of fathers). Individuals who died were coded as event 2; this was not exclusive of event 1 (60.1% of mothers, 73.4% of fathers). The follow-up time between the start of the study (1 January 1986) and each individual event was calculated, using the latest date of a type 2 diabetes diagnosis event (22 November 2020) as the study endpoint.

#### Cumulative incidence curves for parental type 2 diabetes and death

Estimated cumulative incidence curves for type 2 diabetes and death, grouped by BW and PW quartiles (first [lowest] and fourth [highest] only), were built using the ‘cmprsk’ package [20]. The *p* values comparing the subdistribution estimates for each event (type 2 diabetes and death separately) between the first and the fourth quartiles were calculated using the same package. Gray’s test for equality between cumulative incidence functions was used to determine whether there were significant differences in incidence between those quartile curves.

#### Survival analysis of parental type 2 diabetes and death

Different sets of regression models were built, investigating type 2 diabetes diagnosis risk and risk of death as dependent variables separately. The investigation of parental type 2 diabetes diagnosis risk with competing risk of death was performed through Fine–Gray competing risk survival regression analyses, using the ‘cmprsk’ package. The investigation of parental risk of death was performed through Cox survival regression analyses, using the ‘survival’ package [21]. Sex-stratified *Z*-transformed offspring BW, PW, and BW and PW together (BW+PW) were set as three separate sets of different explanatory variables. Three additional sets of models were built, using categorical quartile versions of offspring BW and PW, in order to investigate non-linear associations. The proportional hazards assumption was tested using the scaled Schoenfeld residuals test [22], after building Cox models using the same sets of predictor variables, using the ‘survival’ package. The determination of variables with time-dependent effects was supported by observational investigation of Schoenfeld residuals against failure time plots, also built using the ‘survival’ package. In order to account for any violation of proportionality, variables with time-dependent effects were included in their respective models with additional interactions of those variables with a logarithmic function of time. To correct for parental age at the start of the study, a variable containing the age of each individual at the start of 1986 was included. Additional explanatory variables included offspring GA and parental HBSIMD. The effect size for each covariate was reported as the subdistribution hazard ratio (SHR) for the Fine–Gray models and as the HR for the Cox models with their 95% CIs.

## Results

### Parental type 2 diabetes and death summary statistics

Summary statistics for the distribution of variables of interest in the maternal and paternal datasets are shown in ESM Table 1. A cumulative 15.3% and 17.1% of Walker mothers and fathers, respectively, developed type 2 diabetes, and 61.5% and 74.5% of mothers and fathers, respectively, died. Table 1 shows the percentages of mothers and fathers alive and who developed type 2 diabetes across the decades.

**Table 1:** Summary of living Walker parents across the decades

Cut-off year	Walker mothers (*n*=12,825)				Walker fathers (*n*=7386)			
	Alive	T2D	Age (years)	Age at T2D diagnosis (years)	Alive	T2D	Age (years)	Age at T2D diagnosis (years)
2020	4695 (36.61)	778 (16.57)	84.74 ± 5.67	70.79 ± 8.19	1835 (24.84)	435 (23.71)	85.17 ± 4.97	69.40 ± 8.51
2010	7919 (61.75)	1012 (12.78)	77.03 ± 6.59	70.75 ± 8.45	3786 (51.26)	653 (17.25)	77.67 ± 6.07	69.66 ± 8.62
2000	10,511 (81.96)	521 (4.96)	68.43 ± 7.24	70.15 ± 8.68	5576 (74.49)	389 (6.98)	69.22 ± 6.67	68.96 ± 8.58
1990	12,257 (95.57)	116 (0.95)	59.2 ± 7.62	69.88 ± 8.67	7042 (95.34)	119 (1.69)	60.39 ± 7.21	68.57 ± 8.55

Data are *n* (%) or mean ± SD

Maternal and paternal datasets were analysed separately. The cut-off was set as 1 January for each decade

The ‘Alive’ column shows the number and percentage of living individuals within the total sample. The ‘T2D’ column includes the number and percentage of living individuals who developed type 2 diabetes

T2D, type 2 diabetes

### Differences in offspring birthweight and placental weight by parental type 2 diabetes status

**Fig. 1:**
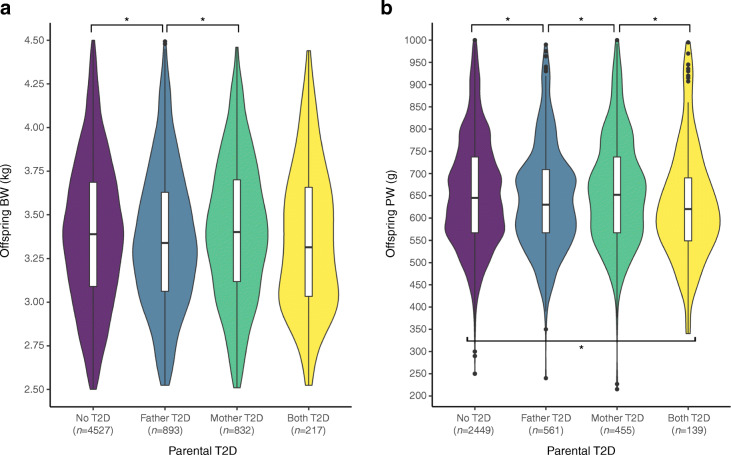
Violin plots of offspring BW (**a**) and PW (**b**) by the later development of parental type 2 diabetes. Vertical box-and-whiskers plots are included. The *p* values for the difference in means between each pair of samples were calculated using Welch two-sample *t* tests (*p<0.05). T2D, type 2 diabetes

Table 2 shows that mean offspring BW from mothers and fathers who developed type 2 diabetes was 11.3 g higher (*p*=0.263) and 36.7 g lower (*p*=0.003) at birth, respectively, than offspring of parents who did not develop diabetes. Mean offspring PW from mothers and fathers who developed type 2 diabetes were 3.3 g higher (*p*=0.450) and 18.2 g lower (*p*<0.001) than placentas from offspring of parents who did not develop diabetes. There were no differences in mean GA between offspring of mothers (*p*=0.388) or offspring of fathers (*p*=0.247) who developed type 2 diabetes and offspring whose parents did not develop diabetes. Figure 1 shows that offspring of parents where only the father developed type 2 diabetes were lighter (*p*=0.016) and had lighter placentas (*p*=0.015) than offspring where neither parent developed diabetes. Offspring of parents where only the mother developed type 2 diabetes were heavier (*p*=0.012) and had heavier placentas (*p*=0.012) than offspring of parents where only the father developed diabetes.

**Table 2:** Summary of variables of interest and differences between parents who developed type 2 diabetes and those who did not

Variable	T2D mothers (*n*=1965)	Undiagnosed mothers (*n*=10,860)	Welch *p* value	T2D fathers (*n*=1266)	Undiagnosed fathers (*n*=6120)	Welch *p* value
Offspring BW (g)	3385.25 ± 410.62	3373.98 ± 410.69	0.263	3358.93 ± 402.32	3395.68 ± 413.37	0.003
Offspring PW (g)	654.67 ± 120.24	651.41 ± 118.30	0.450	639.81 ± 117.88	658.01 ± 120.85	<0.001
Offspring GA (weeks)	39.87 ± 1.33	39.90 ± 1.28	0.388	39.93 ± 1.26	39.88 ± 1.27	0.247
Offspring sex	F: 44.38%; M: 55.62%	F: 43.26%; M: 56.74%	0.360	F: 40.76%; M: 59.24%	F: 40.70%; M: 59.30%	0.971
HBSIMD	2.50 ± 1.46	2.61 ± 1.50	0.001	2.71 ± 1.50	2.71 ± 1.53	0.965
Age in 1986 (years)	52.30 ± 7.01	53.24 ± 7.82	<0.001	52.32 ± 6.45	54.84 ± 7.47	<0.001
Deceased	62.29%	61.33%	0.418	67.85%	75.88%	<0.001

Data are mean ± SD unless indicated otherwise

Maternal and paternal datasets were analysed separately

For the HBSIMD, lower indexes represent higher deprivation levels

T2D, type 2 diabetes

### Cumulative percentages of type 2 diabetes and death by offspring birthweight and placental weight quartiles

**Fig. 2:**
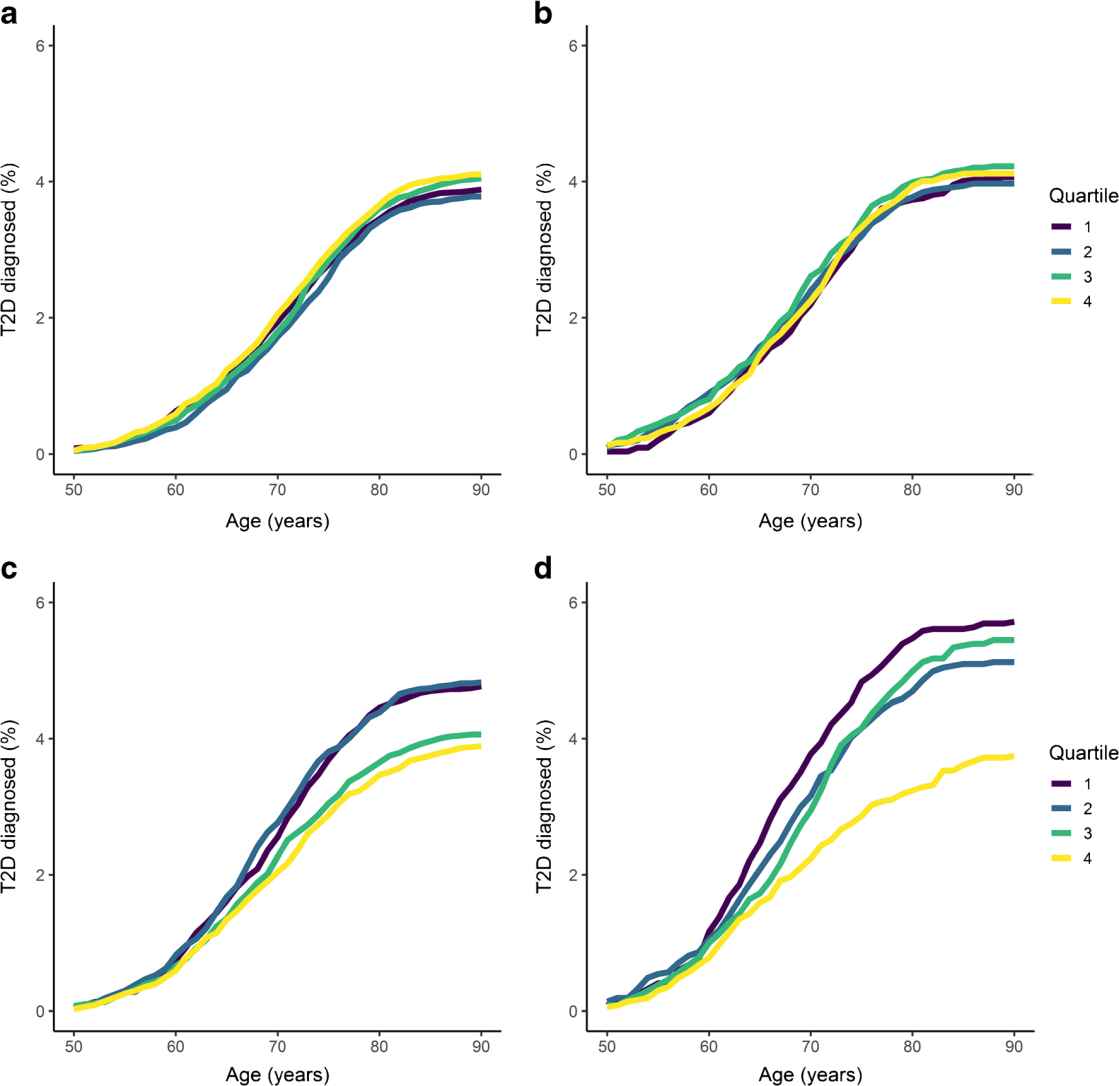
Cumulative percentages of mothers and fathers who developed type 2 diabetes by age and offspring BW and PW quartiles. (**a**, **b**) Cumulative percentage of mothers who developed type 2 diabetes grouped by (**a**) offspring BW and (**b**) offspring PW. (**c**, **d**) Cumulative percentage of fathers who developed type 2 diabetes grouped by (**c**) offspring BW and (**d**) offspring PW. T2D, type 2 diabetes

**Fig. 3:**
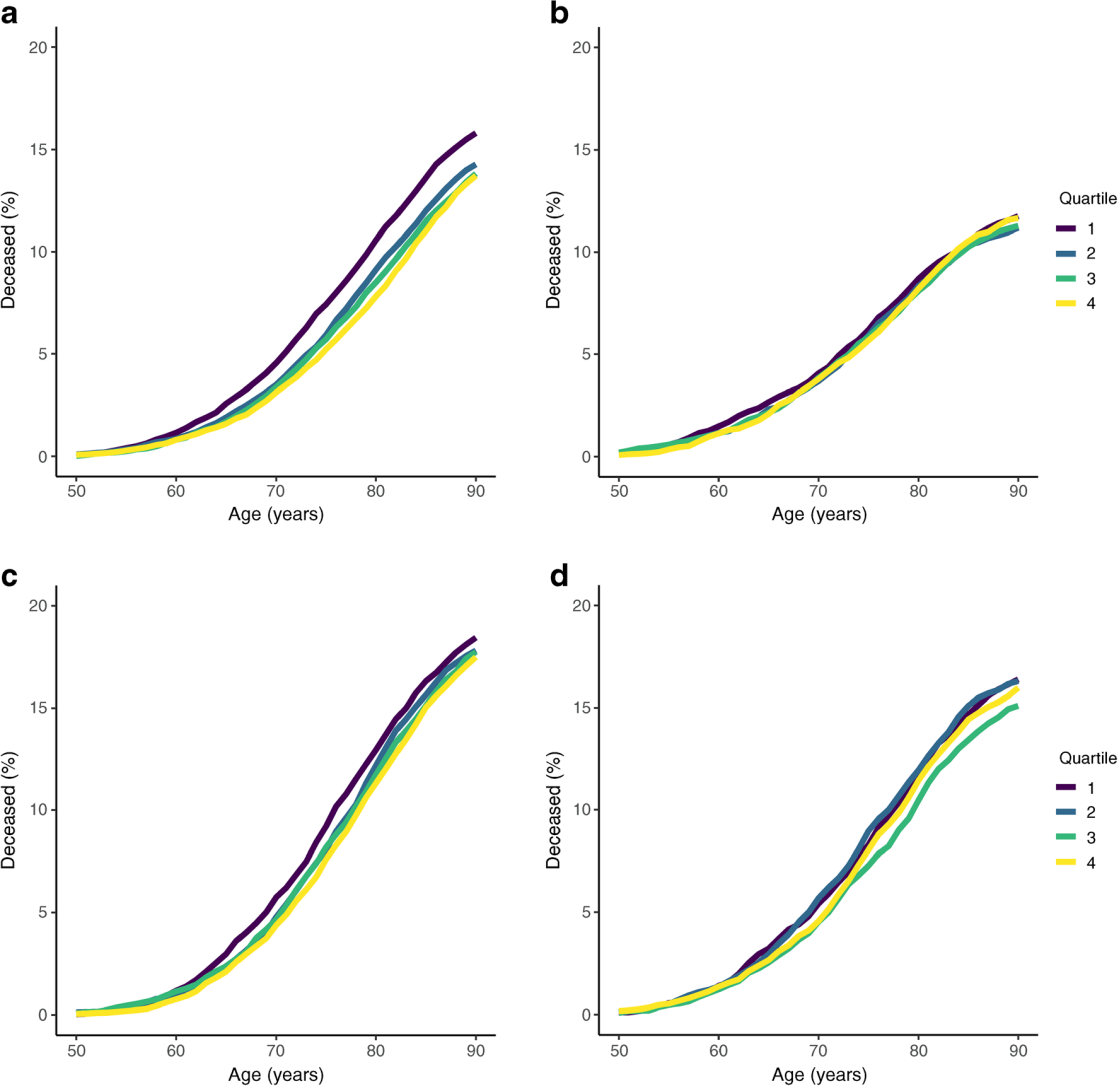
Cumulative percentages of deceased mothers and fathers by age and offspring BW and PW quartiles. (**a**, **b**) Cumulative percentage of deceased mothers grouped by (**a**) offspring BW and (**b**) offspring PW. (**c**, **d**) Cumulative percentage of deceased fathers grouped by (**c**) offspring BW and (**d**) offspring PW

The cumulative percentages of Walker mothers and fathers who developed type 2 diabetes by age and grouped by offspring BW and PW quartiles are displayed in Fig. 2. These plots show visually distinct curves for paternal type 2 diabetes diagnosis by offspring BW and PW quartiles, with lower quartiles generally displaying higher rates of type 2 diabetes diagnosis. In contrast, there was no substantial difference in maternal type 2 diabetes diagnosis rates between offspring BW or PW quartiles. Figure 3 shows the cumulative percentages of deceased mothers and fathers by age, also grouped by offspring BW and PW. Offspring BW and PW quartiles had little impact on paternal mortality. For the mothers, the lowest offspring BW quartile was associated with significantly higher mortality rates than the other quartiles, while offspring PW had no effect.

### Parental summary statistics by offspring BW and PW quartiles

Using the data on the living individuals recorded up to 2010, parental variables were summarised by offspring BW and PW quartiles (Tables 3 and 4). Higher offspring BW and PW quartiles were associated with lower percentages of fathers subsequently diagnosed with type 2 diabetes. On the contrary, there was no significant difference in the development of maternal type 2 diabetes between either offspring BW or offspring PW quartiles. The mortality rate was highest among mothers with offspring in the lowest BW quartile, but no significant difference was found in paternal mortality rate by offspring BW quartile. The mortality rate did not differ by offspring PW quartile for either mothers or fathers.

**Table 3:** Summary of maternal variables of interest by *Z*-transformed offspring BW and PW quartiles

Variable	Walker mothers alive in 2010 (*n*=7919)									
	BW analysis (*n*=1978 per quartile)					PW analysis (*n*=1066 for Q1, Q2, Q3; *n*=1065 for Q4)				
	Q1	Q2	Q3	Q4	ANOVA *p* value	Q1	Q2	Q3	Q4	ANOVA *p* value
Offspring BW (g)	2883.76 ± 160.19	3246.91 ± 90.56	3520.56 ± 100.65	3917.70 ± 202.48	<0.001	3158.66 ± 342.85	3307.15 ± 349.20	3456.77 ± 349.87	3677.24 ± 371.90	<0.001
Offspring PW (g)	580.41 ± 99.23	624.18 ± 97.40	667.66 ± 102.89	738.08 ± 115.18	<0.001	514.45 ± 48.83	602.33 ± 22.20	683.21 ± 22.01	813.50 ± 69.60	<0.001
Offspring GA (weeks)	39.50 ± 1.38	39.87 ± 1.23	40.08 ± 1.18	40.26 ± 1.17	<0.001	39.79 ± 1.28	39.87 ± 1.23	39.96 ± 1.23	40.03 ± 1.25	<0.001
T2D	392 (19.82)	350 (17.69)	377 (19.06)	372 (18.81)	0.392	196 (18.39)	180 (16.89)	194 (18.20)	196 (18.40)	0.765
Age at T2D diagnosis (years)	70.66 ± 8.53	70.75 ± 8.35	70.93 ± 8.53	70.66 ± 8.42	0.968	69.03 ± 8.53	68.25 ± 7.68	68.35 ± 8.20	68.61 ± 8.06	0.790
Deceased	913 (46.16)	891 (45.05)	829 (41.91)	862 (43.58)	0.043	342 (32.08)	338 (31.71)	339 (31.80)	355 (33.33)	0.843
Age at death (years)	82.72 ± 6.62	83.43 ± 6.58	83.96 ± 6.71	84.04 ± 6.70	<0.001	80.34 ± 6.52	79.57 ± 6.56	79.82 ± 6.61	79.76 ± 6.13	0.446
HBSIMD	2.61 ± 1.46	2.74 ± 1.50	2.86 ± 1.51	2.90 ± 1.52	<0.001	2.80 ± 1.50	2.81 ± 1.48	2.87 ± 1.53	2.97 ± 1.52	0.032

Data are mean ± SD or *n* (%)

Only Walker mothers alive at the start of 2010 were included

For the HBSIMD, lower indexes represent higher deprivation levels

Q, quartile; T2D, type 2 diabetes

**Table 4:** Summary of paternal variables of interest by *Z*-transformed offspring BW and PW quartiles

Variable	Walker fathers alive in 2010 (*n*=3786)									
	BW analysis (*n*=946 for Q1, Q2, Q3; *n*=945 for Q4)					PW analysis (*n*=607 for Q1, Q3; *n*=606 for Q2, Q4)				
	Q1	Q2	Q3	Q4	ANOVA *p* value	Q1	Q2	Q3	Q4	ANOVA *p* value
Offspring BW (g)	2889.63 ± 161.45	3259.35 ± 94.96	3524.82 ± 101.98	3928.34 ± 202.42	<0.001	3167.21 ± 344.26	3309.67 ± 334.07	3457.00 ± 347.63	3678.11 ± 378.43	<0.001
Offspring PW (g)	582.43 ± 95.06	631.43 ± 102.31	670.75 ± 108.48	742.41 ± 115.81	<0.001	516.23 ± 47.33	605.85 ± 23.39	685.52 ± 20.98	819.53 ± 72.44	<0.001
Offspring GA (weeks)	39.44 ± 1.38	39.85 ±1.22	40.02 ± 1.16	40.25 ± 1.18	<0.001	39.70 ± 1.29	39.82 ± 1.25	39.95 ± 1.23	49.97 ± 1.23	<0.001
T2D	251 (26.53)	242 (25.58)	222 (23.47)	187 (19.79)	0.003	164 (27.02)	152 (25.08)	165 (27.18)	116 (19.14)	0.003
Age at T2D diagnosis (years)	69.37 ± 8.09	69.57 ± 8.45	69.55 ± 9.30	70.23 ± 8.73	0.761	67.39 ± 7.87	67.99 ± 8.45	69.21 ± 8.19	68.83 ± 9.67	0.217
Deceased	529 (55.92)	512 (54.12)	527 (55.71)	529 (55.98)	0.828	295 (48.60)	303 (50.00)	280 (46.13)	295 (48.68)	0.593
Age at death (years)	81.99 ± 6.34	82.97 ± 5.96	83.19 ± 6.09	83.55 ± 6.12	<0.001	80.96 ± 5.80	80.24 ± 5.82	80.72 ± 5.95	80.99 ± 5.83	0.372
HBSIMD	2.82 ± 1.51	2.91 ± 1.51	3.02 ± 1.51	3.08 ± 1.52	<0.001	3.00 ± 1.51	2.97 ± 1.50	2.99 ± 1.52	3.18 ± 1.49	0.057

Data are mean ± SD or *n* (%)

Only Walker fathers alive at the start of 2010 are included

For the HBSIMD, lower indexes represent higher deprivation levels

Q, quartile; T2D, type 2 diabetes

### Estimated cumulative incidence for type 2 diabetes and death, by offspring BW and PW

**Fig. 4:**
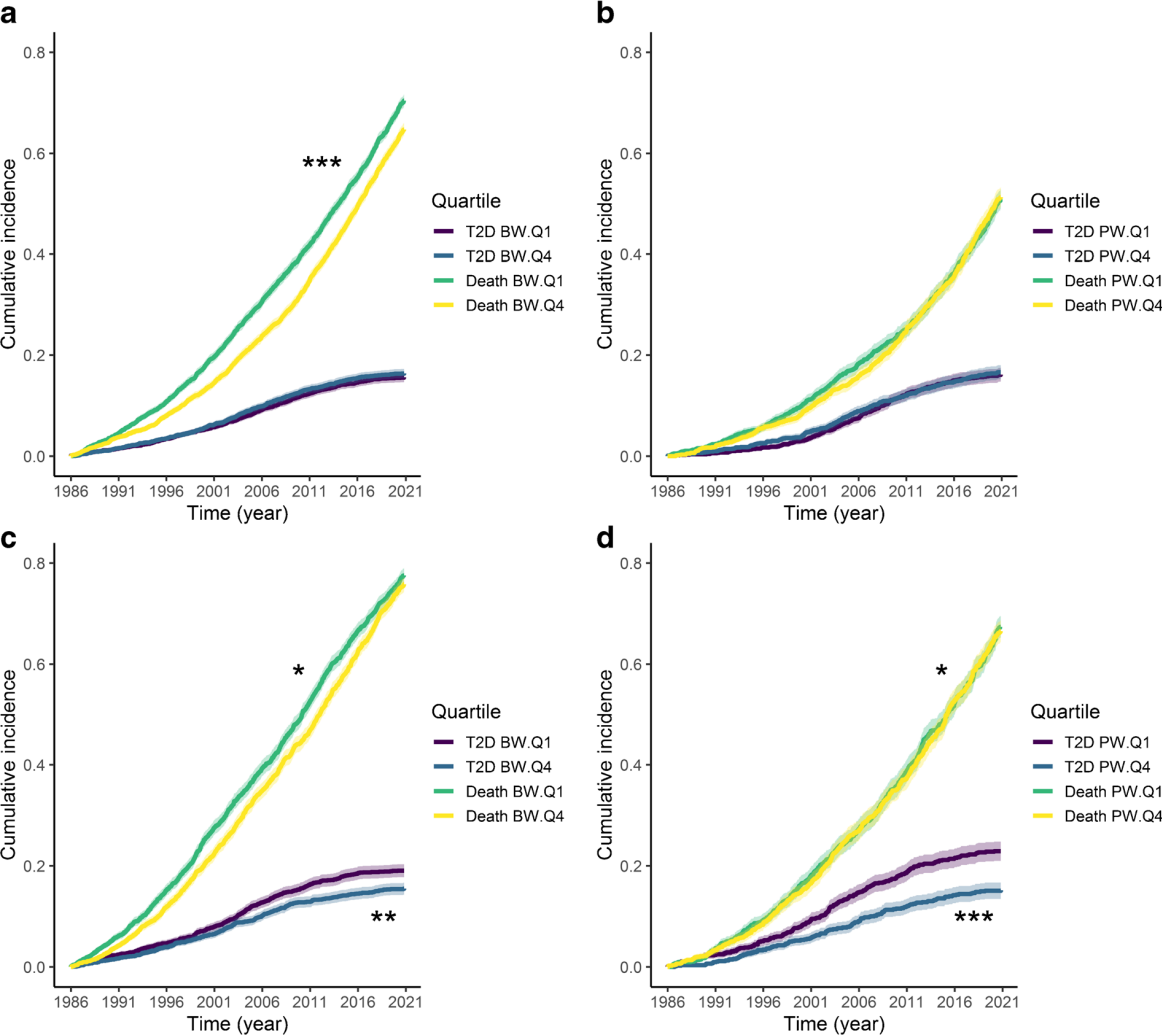
Estimated cumulative incidence curves for maternal and paternal type 2 diabetes and death by age and offspring BW and PW quartiles. Only quartiles 1 and 4 are plotted for clarity. The *p* values were calculated using Gray’s test of equality (*p<0.05, **p<0.01, ***p<0.001). (**a**, **b**) Cumulative maternal incidence of type 2 diabetes and death grouped by (**a**) offspring BW and (**b**) offspring PW. (**c**, **d**) Cumulative paternal incidence of type 2 diabetes and death grouped by (**c**) offspring BW and (**d**) offspring PW. T2D, type 2 diabetes

Estimated cumulative incidence curves for type 2 diabetes and death, grouped by offspring BW and PW quartiles 1 and 4, are displayed in Fig. 4. These plots show a higher incidence of paternal type 2 diabetes for the lowest offspring BW (*p*=0.003) and PW (*p*<0.001) quartiles. The incidence curves for maternal type 2 diabetes are much closer together, with no significant interquartile differences for either the offspring BW (*p*=0.465) or the offspring PW (*p*=0.917) quartiles. The lowest BW quartile was associated with a higher incidence of paternal death for most of its trajectory than the highest BW quartile (*p*=0.021). For BW, the curves for maternal death were very distinct (*p*<0.001), with the lowest quartile displaying a higher cumulative incidence of death. No clear difference in the offspring PW quartile trajectories was found for either the incidence of maternal death (*p*=0.648) or the incidence of paternal death (*p*=0.049).

### Fine–Gray survival analysis of parental type 2 diabetes diagnosis risk

The numerical results from the Fine–Gray survival regression are presented in Tables 5 and 6. Offspring BW and PW were inversely associated with the paternal risk of type 2 diabetes diagnosis, with SHRs of 0.92 (95% CI 0.87, 0.98; *p*=0.005) and 0.87 (95% CI 0.81, 0.94; *p*<0.001), respectively. When investigated together in the same model (BW+PW), the inverse associations with paternal type 2 diabetes remained, but the effect of BW became non-significant (Table 6). Offspring BW, PW and BW+PW combined were not significantly associated with maternal type 2 diabetes risk (Table 5).

**Table 5:** Survival analysis for maternal type 2 diabetes and death

Covariate	Survival analysis of Walker mothers							
	Fine–Gray survival analysis for T2D diagnosis risk				Cox survival analysis for risk of death			
	SHR	95% CI (*α*=0.05)	SE	*p* value	HR	95% CI (*α*=0.05)	SE	*p* value
Analysis including only BW								
Offspring BW	1.038	(0.990, 1.087)	0.024	0.120	0.914	(0.892, 0.936)	0.012	<0.001
Offspring GA	0.857	(0.763, 0.962)	0.059	0.009	0.935	(0.869, 1.005)	0.037	0.068
Age in 1986	1.086	(1.058, 1.115)	0.013	<0.001	1.100	(1.087, 1.113)	0.006	<0.001
HBSIMD	0.943	(0.915, 0.973)	0.016	<0.001	0.722	(0.670, 0.778)	0.038	<0.001
Offspring GA×log(t)	1.051	(1.009, 1.095)	0.021	0.017	1.024	(0.999, 1.050)	0.012	0.052
Age in 1986×log(t)	0.964	(0.955, 0.973)	0.005	<0.001	1.003	(0.999, 1.008)	0.002	0.106
HBSIMD×log(t)	–	–	–	–	1.059	(1.033, 1.085)	0.013	<0.001
Analysis including only PW								
Offspring PW	1.029	(0.962, 1.101)	0.034	0.400	0.998	(0.960, 1.038)	0.020	0.928
Offspring GA	0.990	(0.939, 1.048)	0.028	0.720	0.971	(0.942, 1.002)	0.016	0.066
Age in 1986	1.115	(1.074, 1.158)	0.019	<0.001	1.113	(1.106, 1.120)	0.003	<0.001
HBSIMD	0.923	(0.881, 0.966)	0.023	<0.001	0.846	(0.823, 0.869)	0.014	<0.001
Age in 1986×log(t)	0.960	(0.948, 0.973)	0.007	<0.001	–	–	–	–
Analysis including BW and PW								
Offspring BW	1.066	(0.982, 1.158)	0.042	0.130	0.846	(0.806, 0.887)	0.025	<0.001
Offspring PW	0.997	(0.922, 1.079)	0.040	0.950	1.083	(1.035, 1.133)	0.023	<0.001
Offspring GA	0.980	(0.926, 1.038)	0.029	0.490	0.996	(0.965, 1.028)	0.016	0.817
Age in 1986	1.115	(1.074, 1.158)	0.019	<0.001	1.115	(1.108, 1.122)	0.003	<0.001
HBSIMD	0.920	(0.878, 0.963)	0.023	<0.001	0.852	(0.829, 0.875)	0.014	<0.001
Age in 1986×log(t)	0.960	(0.948, 0.973)	0.007	<0.001	–	–	–	–

The analysis of type 2 diabetes risk accounted for the competing risk of death

For the HBSIMD, lower indexes represent higher deprivation levels

T2D, type 2 diabetes

**Table 6:** Survival analysis for paternal type 2 diabetes and death

Covariate	Survival analysis of Walker fathers							
	Fine–Gray survival analysis for T2D diagnosis risk				Cox survival analysis for risk of death			
	SHR	95% CI (*α*=0.05)	SE	*p* value	HR	95% CI (*α*=0.05)	SE	*p* value
Analysis including only BW								
Offspring BW	0.919	(0.867, 0.975)	0.030	0.005	0.947	(0.920, 0.975)	0.015	<0.001
Offspring GA	1.039	(0.993, 1.087)	0.023	0.097	1.020	(0.997, 1.042)	0.011	0.086
Age in 1986	1.051	(1.026, 1.078)	0.013	<0.001	1.110	(1.105, 1.115)	0.002	<0.001
HBSIMD	0.989	(0.953, 1.026)	0.019	0.550	0.721	(0.667, 0.778)	0.039	<0.001
Age in 1986×log(t)	0.963	(0.954, 0.972)	0.005	<0.001	–	–	–	–
HBSIMD×log(t)	–	–	–	–	1.070	(1.043, 1.098)	0.013	<0.001
Analysis including only PW								
Offspring PW	0.872	(0.808, 0.941)	0.039	<0.001	0.984	(0.944, 1.025)	0.021	0.429
Offspring GA	1.027	(0.968, 1.089)	0.030	0.380	0.865	(0.759, 0.987)	0.067	0.031
Age in 1986	1.096	(1.066, 1.125)	0.014	<0.001	1.114	(1.106, 1.121)	0.003	<0.001
HBSIMD	0.978	(0.933, 1.025)	0.024	0.350	0.669	(0.592, 0.758)	0.063	<0.001
Offspring GA×log(t)	–	–	–	–	1.062	(1.016, 1.109)	0.022	0.008
Age in 1986×log(t)	0.955	(0.946, 0.965)	0.005	<0.001	–	–	–	–
HBSIMD×log(t)	–	–	–	–	1.092	(1.048, 1.138)	0.021	<0.001
Analysis including BW and PW								
Offspring BW	0.962	(0.880, 1.051)	0.045	0.390	0.941	(0.895, 0.989)	0.025	0.016
Offspring PW	0.888	(0.814, 0.970)	0.045	0.008	1.014	(0.967, 1.063)	0.024	0.566
Offspring GA	1.031	(0.971, 1.095)	0.031	0.320	0.870	(0.763, 0.992)	0.067	0.038
Age in 1986	1.096	(1.067, 1.126)	0.014	<0.001	1.114	(1.107, 1.122)	0.003	<0.001
HBSIMD	0.978	(0.933, 1.025)	0.024	0.350	0.669	(0.591, 0.758)	0.063	<0.001
Offspring GA×log(t)	–	–	–	–	1.063	(1.018, 1.111)	0.022	0.006
Age in 1986×log(t)	0.955	(0.946, 0.965)	0.005	<0.001	–	–	–	–
HBSIMD×log(t)	–	–	–	–	1.093	(1.049, 1.149)	0.021	<0.001

The analysis of type 2 diabetes risk accounted for the competing risk of death

For the HBSIMD, lower indexes represent higher deprivation levels

T2D, type 2 diabetes

The sensitivity analyses including all term births showed similar results (ESM Tables 2 and 3) but identified associations between maternal risk of type 2 diabetes and offspring BW (SHR 1.06 [95% CI 1.01, 1.11]; *p*=0.011).

### Cox survival analysis of parental risk of death

The numerical results from the Cox survival regression analyses are presented in Tables 5 and 6. Offspring BW was inversely associated with maternal and paternal risk of death, with HRs of 0.91 (95% CI 0.89, 0.94; *p*<0.001) and 0.95 (95% CI 0.92, 0.98; *p*<0.001), respectively. Offspring PW was not significantly associated with maternal or paternal risk of death. When included simultaneously in the same model using the maternal dataset, offspring BW and PW showed diverging but significant HRs: offspring BW was inversely associated with maternal risk of death (HR 0.85 [95% CI 0.81, 0.89]; *p*<0.001), while offspring PW showed positive associations (HR 1.08 [95% CI 1.04, 1.13]; *p*<0.001).

Fine–Gray and Cox analyses conducted using categorical quartiles of offspring BW and PW showed similar results (ESM Tables 4 and 5), as did the sensitivity analyses including all term births (ESM Tables 2 and 3).

## Discussion

Our study represents the first investigation of paternal type 2 diabetes and risk of death modelled by offspring BW and PW and provides novel epidemiological evidence supporting the association between parental type 2 diabetes and offspring fetal growth. The Walker cohort [14] represents a unique dataset to explore these relationships, capturing a vast breadth of gestational information and birth measurements with the ability to link them to national healthcare datasets to obtain the parents’ health records, an ageing population with significant morbidity and mortality.

Paternal type 2 diabetes was associated with offspring BW independently and in an opposite direction to that for maternal type 2 diabetes, consistent with previous studies [9, 13]. BW was lower in offspring of fathers who later developed type 2 diabetes than in offspring of fathers who did not develop type 2 diabetes, as previously reported [9, 10, 13]. In contrast, BW was higher in offspring of mothers who later developed type 2 diabetes than in offspring of mothers who did not (although the difference was not statistically significant), as others have shown [7, 9, 10, 13, 23, 24]. In a novel approach, we examined the association between offspring BW and parental risk of developing type 2 diabetes through Fine–Gray survival regression, accounting for the competing risk of overall death. We demonstrated small but significant associations between offspring BW and paternal type 2 diabetes risk: an increase of 1 SD in the offspring BW *Z* score was associated with an 8.1% decrease in the SH for type 2 diabetes in fathers. Similar associations have been previously reported [9, 11, 12, 25, 26]. In addition, offspring BW was inversely associated with parental mortality risk, with an increase of 1 SD in the offspring BW *Z* score accounting for an 8.6% and 5.3% reduction in the maternal and paternal risk of death, respectively, at any given time. This relationship, also previously reported [27, 28], is likely to be driven by maternal health and its effect on fetal growth, with the paternal association most likely being a reflection of the shared parental environment. This would also explain the reduced association of offspring BW with paternal mortality compared with the association with maternal mortality.

Our study extends previous observations of the relationship between BW and parental diabetes to consider effects of the placenta. While correlations between BW and PW have been described previously [23, 29, 30], this is the first time to our knowledge that offspring PW has been investigated in relation to the type 2 diabetes status of both mothers and fathers. We found that offspring of fathers who were later diagnosed with type 2 diabetes had placentas that were 18 g lighter than offspring of fathers who did not develop diabetes. We found no significant relationship between PW and subsequent type 2 diabetes in mothers, although our findings may have been limited by sample size, particularly as other studies have reported associations between maternal gestational diabetes and increased placental growth [29, 30]. This is also the first study providing evidence of increased offspring PW being associated with a reduced risk of a later type 2 diabetes diagnosis in fathers, with an increase of 1 SD in offspring PW *Z* score being associated with a 12.8% decrease in the SH of paternal type 2 diabetes. These observations suggest that the determinants of PW behave similarly to those of BW, but also parallel the original findings of Hattersley et al on the transmission of the glucokinase gene and the differential effects of maternal and fetal diabetes [7]. The associations found are likely reflecting the repercussions of common genetic variants associated with type 2 diabetes on offspring BW and PW: maternal diabetes increases fetal growth through the action of mothers’ own genotypes, increasing glucose levels in utero, while paternal diabetes reduces fetal growth when fathers’ type 2 diabetes susceptibility alleles are inherited by their offspring through impaired fetal glucose metabolism, which leads to impaired fetal and placental growth.

It is evident that parental genes differentially expressed by the fetus perform significant roles in regulating fetal growth and placental function [31–33]. Paternal genes have also been reported to contribute to maternal gestational conditions such as pre-eclampsia [34] through genetic actions of the fetus. Following this rationale, we suggest that the observed association between offspring PW and paternal type 2 diabetes is also a result of the offspring’s type 2 diabetes predisposition alleles inherited from the father, culminating in alteration of placental growth. This association may be caused by direct gene expression in the placenta or by reduced fetal insulin secretion, influencing both BW and PW [35]. As fetal growth is heavily regulated by insulin, BW may be acting as a mediator of PW, obscuring any direct relationship between parental diabetes and offspring PW. When BW and PW were analysed together in the same survival model, the effect of PW was attenuated, suggesting that the association between offspring PW and parental type 2 diabetes may be mediated by fetal insulin dysfunction alleles.

While the link between PW and paternal mortality has not been investigated previously, associations between increased offspring PW (and its ratio to BW) and maternal mortality were identified in a US cohort of over 33,000 pregnancies from the 1960s [36]. We also found associations between offspring PW and maternal mortality when adjusted for BW, with an 8.3% increase in the maternal risk of death for every increase of 1 SD in the PW *Z* score, for any given BW, which probably reflects placental inefficiency. Maternal vasculopathies are associated with poor placental perfusion [37], which may explain the link between maternal health and placental insufficiency. We found no association between PW and paternal risk of death.

A strength of our study is the use of the Walker cohort, a valuable and untapped resource containing the longitudinal records of an ageing industrialised post-World War II population. We linked to SCI-Diabetes, the national diabetes register, in which type of diabetes is recorded by clinicians. The accuracy of this variable is improved using an algorithm that combines information from the clinician-recorded diabetes type variable and prescription data [38, 39]. Diabetes data were not regularly collected before 1986, which might have resulted in earlier diagnoses being missed, but these diagnoses were probably introduced with a delay into the records. Other limitations include the lack of data on parental smoking status or other substance abuse, which prevented us from adjusting the models for the recognisable effect of such lifestyles [40]. The HBSIMD was used as a proxy variable because of the lack of socioeconomic class data at the time of offspring birth. However, the HBSIMD was highly correlated with a social class categorisation [41] performed on the parental occupation data recorded in the Walker cohort (analyses not shown). Parental height or weight measurements were also largely missing. PW measurements started being recorded during the course of the cohort, which halved the sample size for the analyses including PW, limiting statistical power. The SHRs resulting from Fine–Gray analyses do not allow for a straightforward assessment and comparison of the magnitudes of the effects studied [42], being indicative of hazard rates but not incidence or risk. The proportional hazards assumption for the Fine–Gray models was tested using the scaled Schoenfeld test over residuals from Cox models. Other approaches might have been more appropriate, but this test represented a simpler and more replicable method. Finally, any covariates violating the proportional hazards assumption in the Cox or Fine–Gray models were adjusted through a log×time interaction term. This inclusion of additional covariate×time interaction terms increased the complexity of the model and thus the risk of overfitting; however, this was deemed necessary to deal with the more crucial proportional hazards assumption.

In conclusion, we found novel associations between reduced offspring BW and PW and paternal type 2 diabetes risk. We believe the observed associations reflect the inheritance of type 2 diabetes susceptibility variants and the interplay of maternal and fetal glucose metabolism in utero, as described by the fetal insulin hypothesis. To understand the relationship between offspring PW and parental type 2 diabetes, additional research is required on the particular effects of the inherited type 2 diabetes variants on placental growth, including how this might be influenced by intrauterine hyperglycaemia. These results provide new insights into the link between genetic type 2 diabetes predisposition and offspring birth outcomes.

## Supplementary information

ESM (PDF 320 kb)

## Data Availability

The datasets generated and analysed during the current study are not publicly available because of the need for ethics and regulatory approvals but are available from the corresponding author on reasonable request.

## Abbreviations

BW: Birthweight
DOB: Date of birth
GA: Gestational age
GDM: Gestational diabetes mellitus
GWA: Genome-wide association
HBSIMD: Health board-specific Scottish Index of Multiple Deprivation
PW: Placental weight
SH: Subdistribution hazard
SHR: Subdistribution hazard ratio
