# Supplementary material for: Associations between parental type 2 diabetes risk and offspring birthweight and placental weight: a survival analysis using the Walker cohort
Source: Diabetologia. 2022 Aug 11;65(12):2084–97. doi: 10.1007/s00125-022-05776-5 (PMC9630220; doi:10.1007/s00125-022-05776-5)
Supplement: Supplementary file 1 — (PDF 320 kb) [file 125_2022_5776_MOESM1_ESM.pdf]

	Maternal Dataset (n=12825)	Missing	Paternal Dataset (n=7386)	Missing	Welch *p* value
**Offspring BW (g)**	3376.91 ± 411.28	15 (0.12%)	3389.36 ± 410.93	8 (0.11%)	0.026
**Offspring PW (g)**	651.52 ± 118.26	7026 (54.78%)	653.83 ± 120.04	3526 (47.73%)	0.342
**Offspring GA (weeks)**	39.89 ± 1.28	318 (2.48%)	39.89 ± 1.27	188 (2.55%)	0.901
**Offspring sex**	F: 5570 (43.43%)<br>M: 7255 (56.57%)	0%	F: 3007 (40.71%)<br>M: 4379 (59.29%)	0%	<0.001
**T2D**	1965 (15.32%)	-	1266 (17.14%)	-	-
**Age at T2D diagnosis (years)**	69.88 ± 8.66	209 (1.63%)	68.57 ± 8.55	101 (1.37%)	<0.001
**Cumulative Deceased**	7884 (61.47%)	-	5503 (74.50%)	-	-
**Age at Death (years)**	77.36 ± 9.91	209 (1.63%)	76.14 ± 9.31	101 (1.37%)	<0.001
**HBSIMD**	1: 4120 (33.49%)<br>2: 2719 (22.10%)<br>3: 1532 (12.45%)<br>4: 1867 (15.18%)<br>5: 2063 (16.77%)	524 (4.08%)	1: 2233 (31.16%)<br>2: 1519 (21.19%)<br>3: 901 (12.57%)<br>4: 1104 (15.41%)<br>5: 1409 (19.66%)	220 (2.98%)	<0.001

ESM Table 1. Summary statistics for the maternal and paternal datasets. Data are *n* (%) or mean ± SD. The *Missing* column next to each dataset refers to the number missing records for each measurement. The *Missing* value for the ages at type 2 diabetes diagnosis and death represent the percentage of individuals missing date of birth. For the *HBSIMD*, lower indexes represent higher deprivation levels. T2D, type 2 diabetes.

	Survival Analysis of Walker Mothers (all term births, n=13,902)							
	**Fine-Gray survival analysis for T2D diagnosis risk**				**Cox survival analysis for risk of death**			
	**SHR**	**95% CI (α=0.05)**	**SE**	***p* value**	**HR**	**95% CI (α=0.05)**	**SE**	***p* value**
	Analysis including only BW							
**Offspring BW**	1.062	(1.014, 1.112)	0.024	0.011	0.822	(0.750, 0.901)	0.047	<0.001
**Offspring GA**	0.860	(0.775, 0.954)	0.053	0.004	0.931	(0.868, 0.998)	0.036	0.045
**Age in 1986**	1.073	(1.040, 1.107)	0.016	<0.001	1.099	(1.086, 1.111)	0.006	<0.001
**HBSIMD**	0.940	(0.912, 0.968)	0.015	<0.001	0.721	(0.671, 0.775)	0.036	<0.001
**Offspring BW × log(t)**	-	-	-	-	1.030	(0.998, 1.062)	0.016	0.065
**Offspring GA × log(t)**	1.049	(1.012, 1.088)	0.019	0.010	1.025	(1.001, 1.050)	0.012	0.038
**Age in 1986 × log(t)**	0.969	(0.958, 0.980)	0.006	<0.001	1.005	(1.001, 1.009)	0.002	0.012
**HBSIMD × log(t)**	-	-	-	-	1-062	(1.037, 1.087)	0.012	<0.001
	Analysis including only PW							
**Offspring PW**	1.051	(0.985, 1.121)	0.033	0.140	0.989	(0.952, 1.026)	0.019	0.549
**Offspring GA**	0.993	(0.941, 1.047)	0.027	0.790	0.963	(0.935, 0.993)	0.015	0.014
**Age in 1986**	1.105	(1.061, 1.151)	0.021	<0.001	1.113	(1.106, 1.120)	0.003	< 0.001
**HBSIMD**	0.923	(0.883, 0.965)	0.023	<0.001	0.846	(0.824, 0.869)	0.013	< 0.001
**Age 1986 × log(t)**	0.965	(0.951, 0.978)	0.007	<0.001	-	-	-	-
	Analysis including BW and PW							
**Offspring BW**	1.054	(0.968, 1.148)	0.043	0.023	0.832	(0.792, 0.874)	0.025	<0.001
**Offspring PW**	1.023	(0.945, 1.106)	0.040	0.580	1.085	(1.037, 1.134)	0.023	<0.001
**Offspring GA**	0.985	(0.931, 1.041)	0.028	0.580	0.991	(0.961, 1.022)	0.016	0.564
**Age in 1986**	1.105	(1.061, 1.151)	0.021	<0.001	1.115	(1.108, 1.121)	0.003	<0.001
**HBSIMD**	0.921	(0.881, 0.963)	0.023	<0.001	0.853	(0.831, 0.876)	0.013	<0.001
**Age in 1986 × log(t)**	0.964	(0.951, 0.978)	0.007	<0.001	-	-	-	-

ESM Table 2. Survival analysis for maternal type 2 diabetes and death including all term births. Analyses performed using wider BW (<900g or >6,5kg) and PW (<200g or >1,500g) inclusion ranges. The analysis of type 2 diabetes risk accounted for the competing risk of death. For the HBSIMD, lower indexes represent increased deprivation levels. T2D, type 2 diabetes.

	Survival Analysis of Walker Fathers (all term births, n=8,002)							
	**Fine-Gray survival analysis for T2D diagnosis risk**				**Cox survival analysis for risk of death**			
	**SHR**	**95% CI (α=0.05)**	**SE**	***p* value**	**HR**	**95% CI (α=0.05)**	**SE**	***p* value**
	Analysis including only BW							
**Offspring BW**	0.912	(0.860, 0.968)	0.030	0.002	0.949	(0.922, 0.976)	0.014	<0.001
**Offspring GA**	1.037	(0.993, 1.984)	0.022	0.100	1.015	(0.994, 1.037)	0.011	0.159
**Age in 1986**	1.042	(1.011, 1.075)	0.016	0.009	1.112	(1.108, 1.117)	0.002	<0.001
**HBSIMD**	0.981	(0.946, 1.017)	0.018	0.290	0.716	(0.664, 0.772)	0.038	<0.001
**Age in 1986 × log(t)**	0.967	(0.956, 0.979)	0.006	<0.001	-	-	-	-
**HBSIMD × log(t)**	-	-	-	-	1.073	(1.046, 1.100)	0.013	<0.001
	Analysis including only PW							
**Offspring PW**	0.883	(0.817, 0.954)	0.040	0.002	0.988	(0.950, 1.027)	0.020	0.533
**Offspring GA**	1.010	(0.955, 1.069)	0.029	0.730	0.860	(0.757, 0.977)	0.065	0.021
**Age in 1986**	1.094	(1.062, 1.127)	0.015	<0.001	1.114	(1.107, 1.121)	0.003	<0.001
**HBSIMD**	0.970	(0.927, 1.015)	0.023	0.190	0.659	(0.583, 0.744)	0.062	<0.001
**Offspring GA × log(t)**	-	-	-	-	1.063	(1.018, 1.109)	0.022	0.005
**Age in 1986 × log(t)**	0.956	(0.946, 0.967)	0.006	<0.001	-	-	-	-
**HBSIMD × log(t)**	-	-	-	-	1.098	(1.055, 1.143)	0.021	<0.001
	Analysis including BW and PW							
**Offspring BW**	0.909	(0.828, 0.998)	0.048	0.045	0.962	(0.915, 1.010)	0.025	0.122
**Offspring PW**	0.928	(0.846, 1.019)	0.047	0.120	1.008	(0.962, 1.056)	0.024	0.736
**Offspring GA**	1.024	(0.966, 1.084)	0.029	0.430	0.862	(0.759, 0.979)	0.065	0.023
**Age in 1986**	1.095	(1.063, 1.128)	0.015	<0.001	1.115	(1.108, 1.122)	0.003	<0.001
**HBSIMD**	0.973	(0.929, 1.018)	0.023	0.230	0.659	(0.583, 0.744)	0.062	<0.001
**Offspring GA × log(t)**	-	-	-	-	1.064	(1.020, 1.111)	0.022	0.004
**Age in 1986 × log(t)**	0.956	(0.956, 0.967)	0.006	<0.001	-	-	-	-
**HBSIMD × log(t)**	-	-	-	-	1.099	(1.056, 1.144)	0.021	<0.001

ESM Table 3. Survival analysis for paternal type 2 diabetes and death including all term births. Analyses performed using wider BW (<900g or >6,5kg) and PW (<200g or >1,500g) inclusion ranges. The analysis of type 2 diabetes risk accounted for the competing risk of death. For the HBSIMD, lower indexes represent increased deprivation levels. T2D, type 2 diabetes

	Survival Analysis of Walker Mothers (quartile BW & PW)							
	Fine-Gray survival analysis for T2D diagnosis risk				Cox survival analysis for risk of death			
	**SHR**	**95 % CI (α=0.05)**	**SE**	***p* value**	**HR**	**95% CI (α=0.05)**	**SE**	***p* value**
	Analysis including only BW							
**Offspring BW Q1**	1	Baseline Category			1	Baseline Category		
**Offspring BW Q2**	0.961	(0.843, 1.095)	0.067	0.550	0.839	(0.787, 0.894)	0.032	<0.001
**Offspring BW Q3**	1.070	(0.940, 1.219)	0.066	0.310	0.785	(0.736, 0.838)	0.033	<0.001
**Offspring BW Q4**	1.075	(0.943, 1.226)	0.067	0.280	0.770	(0.721, 0.822)	0.033	<0.001
**Offspring GA**	0.858	(0.765, 0.964)	0.059	0.010	0.936	(0.871, 1.007)	0.037	0.075
**Age in 1986**	1.088	(1.059, 1.117)	0.013	<0.001	1.099	(1.086, 1.112)	0.006	<0.001
**HBSIMD**	0.943	(0.914, 0.972)	0.016	<0.001	0.712	(0.661, 0.768)	0.038	<0.001
**Offspring GA × log(t)**	1.051	(1.009, 1.095)	0.021	0.016	1.024	(0.999, 1.049)	0.012	0.055
**Age in 1986 × log(t)**	0.964	(0.955, 0.973)	0.005	<0.001	1.005	(1.001, 1.009)	0.002	0.018
**HBSIMD × log(t)**	-	-	-	-	1.065	(1.039, 1.092)	0.013	<0.001
	Analysis including only PW							
**Offspring PW Q1**	1	Baseline Category			1	Baseline Category		
**Offspring PW Q2**	0.982	(0.812, 1.186)	0.097	0.850	1.001	(0.896, 1.118)	0.056	0.992
**Offspring PW Q3**	1.024	(0.849, 1.235)	0.096	0.810	0.981	(0.879, 1.096)	0.056	0.736
**Offspring PW Q4**	1.064	(0.882, 1.285)	0.096	0.520	1.017	(0.911, 1.135)	0.056	0.765
**Offspring GA**	0.992	(0.939, 1.048)	0.027	0.780	0.971	(0.941, 1.001)	0.016	0.062
**Age in 1986**	1.117	(1.076, 1.160)	0.019	<0.001	1.113	(1.106, 1.120)	0.003	<0.001
**HBSIMD**	0.924	(0.883, 0.968)	0.023	<0.001	0.846	(0.823, 0.869)	0.014	<0.001
**Age in 1986 × log(t)**	0.960	(0.948, 1.973)	0.007	<0.001	-	-	-	-
	Analysis including BW and PW							
**Offspring BW Q1**	1	Baseline Category			1	Baseline Category		
**Offspring BW Q2**	0.993	(0.811, 1.216)	0.103	0.940	0.740	(0.663, 0.826)	0.056	<0.001
**Offspring BW Q3**	1.094	(0.888, 1.346)	0.106	0.400	0.685	(0.610, 0.770)	0.059	<0.001
**Offspring BW Q4**	1.131	(0.907, 1.410)	0.113	0.380	0.635	(0.560, 0.720)	0.064	<0.001
**Offspring PW Q1**	1	Baseline Category			1	Baseline Category		
**Offspring PW Q2**	0.966	(0.796, 1.172)	0.099	0.730	1.076	(0.961, 1.203)	0.057	0.203
**Offspring PW Q3**	0.985	(0.806, 1.204)	0.102	0.880	1.125	(1.001, 1.263)	0.059	0.048
**Offspring PW Q4**	1.001	(0.813, 1.232)	0.106	0.990	1.232	(1.088, 1.394)	0.063	<0.001
**Offspring GA**	0.985	(0.930, 1.043)	0.029	0.600	0.995	(0.964, 1.027)	0.016	0.760
**Age in 1986**	1.117	(0.076, 1.160)	0.019	<0.001	1.115	(1.108, 1.122)	0.003	<0.001
**HBSIMD**	0.922	(0.880, 0.965)	0.023	< 0.001	0.850	(0.828, 0.874)	0.014	<0.001
**Age ’86 × log(t)**	0.960	(0.948, 0.973)	0.007	< 0.001	-	-	-	-

ESM Table 4. Survival analysis for maternal type 2 diabetes and death (categorical BW and PW). Analyses performed using categorical quartile versions of BW and PW. The analysis of type 2 diabetes risk accounted for the competing risk of death. For the HBSIMD, lower indexes represent increased deprivation levels. T2D, type 2 diabetes

	Survival Analysis of Walker Fathers (categorical BW & PW)							
	Fine-Gray survival analysis for T2D diagnosis risk				Cox survival analysis for risk of death			
	SHR	95% CI (α=0.05)	SE	*p* value	HR	95% CI (α=0.05)	SE	*p* value
	Analysis including only BW							
**Offspring BW Q1**	1	Baseline Category			1	Baseline Category		
**Offspring BW Q2**	0.965	(0.828, 1.123)	0.078	0.640	0.920	(0.853, 0.993)	0.039	0.033
**Offspring BW Q3**	0.856	(0.729 1.005)	0.082	0.057	0.886	(0.820, 0.957)	0.040	0.002
**Offspring BW Q4**	0.752	(0.636, 0.889)	0.085	<0.001	0.871	(0.805, 0.942)	0.040	<0.001
**Offspring GA**	1.039	(0.993, 1.088)	0.023	0.093	1.015	(0.994, 1.038)	0.011	0.167
**Age in 1986**	1.053	(1.028, 1.080)	0.013	<0.001	1.111	(1.107, 1.116)	0.002	<0.001
**HBSIMD**	0.987	(0.952, 1.024)	0.019	0.500	0.714	(0.661, 0.771)	0.039	<0.001
**Age in 1986 × log(t)**	0.963	(0.955, 0.972)	0.005	<0.001	-	-	-	-
**HBSIMD × log(t)**	-	-	-	-	1.073	(1.045, 1.101)	0.013	<0.001
	Analysis including only PW							
**Offspring PW Q1**	1	Baseline Category			1	Baseline Category		
**Offspring PW Q2**	0.889	(0.729, 1.083)	0.101	0.240	1.037	(0.928, 1.159)	0.057	0.520
**Offspring PW Q3**	0.905	(0.744, 1.101)	0.100	0.320	0.886	(0.790, 0.992)	0.058	0.036
**Offspring PW Q4**	0.664	(0.535, 0.825)	0.111	<0.001	0.990	(0.884, 1.109)	0.058	0.869
**Offspring GA**	1.022	(0.964, 1.085)	0.030	0.460	0.861	(0.755, 0.981)	0.067	0.024
**Age in 1986**	1.097	(1.068, 1.127)	0.014	<0.001	1.114	(1.107, 1.121)	0.003	<0.001
**HBSIMD**	0.976	(0.932, 1.023)	0.024	0.320	0.663	(0.586, 0.750)	0.063	<0.001
**Offspring GA × log(t)**	-	-	-	-	1.061	(1.016, 1.109)	0.022	0.007
**Age in 1986 × log(t)**	0.955	(0.946, 0.965)	0.005	<0.001	-	-	-	-
**HBSIMD × log(t)**	-	-	-	-	1.094	(1.050, 1.140)	0.021	<0.001
	Analysis including BW and PW							
**Offspring BW Q1**	1	Baseline Category			1	Baseline Category		
**Offspring BW Q2**	1.025	(0.842, 1.248)	0.100	0.800	0.910	(0.811, 1.022)	0.059	0.112
**Offspring BW Q3**	0.847	(0.685, 1.049)	0.109	0.130	0.847	(0.749, 0.956)	0.062	0.007
**Offspring BW Q4**	0.829	(0.655, 1.049)	0.120	0.120	0.891	(0.782, 1.014)	0.066	0.081
**Offspring PW Q1**	1	Baseline Category			1	Baseline Category		
**Offspring PW Q2**	0.910	(0.746, 1.108)	0.101	0.350	1.061	(0.948, 1.187)	0.058	0.305
**Offspring PW Q3**	0.956	(0.781, 1.171)	0.103	0.660	0.920	(0.817, 1.036)	0.060	0.167
**Offspring PW Q4**	0.731	(0.578, 0.925)	0.120	0.009	1.038	(0.915, 1.179)	0.065	0.559
**Offspring GA**	1.033	(0.973, 1.097)	0.031	0.290	1.033	(0.999, 1.068)	0.017	0.055
**Age in 1986**	1.098	(1.069, 1.128)	0.014	<0.001	1.114	(1.107, 1.121)	0.003	<0.001
**HBSIMD**	0.979	(0.934, 1.026)	0.024	0.380	0.666	(0.588, 0.753)	0.063	<0.001
**Age in 1986 × log(t)**	0.956	(0.946, 0.965)	0.005	<0.001	-	-	-	-
**HBSIMD × log(t)**	-	-	-	-	1.093	(1.049, 1.138)	0.021	<0.001

ESM Table 5. Survival analysis for paternal type 2 diabetes and death (categorical BW and PW). Analyses performed using categorical quartile versions of BW and PW. The analysis of type 2 diabetes risk accounted for the competing risk of death. For the HBSIMD, lower indexes represent increased deprivation levels. T2D, type 2 diabetes
